# Estimating the risk of incident SARS-CoV-2 infection among healthcare workers in quarantine hospitals: the Egyptian example

**DOI:** 10.1038/s41598-022-23428-x

**Published:** 2022-11-17

**Authors:** Sofía Jijón, Ahmad Al Shafie, Essam Hassan, EMAE-MESuRS working group on nosocomial SARS-CoV-2 modeling, Audrey Duval, Kenza Hamzi, Niels Hendrickx, Ajmal Oodally, Lulla Opatowski, George Shirreff, David R. M. Smith, Cynthia Tamandjou, Sofía Jijón, Laura Temime, Kévin Jean, Laura Temime, Kévin Jean, Mohamed El-Kassas

**Affiliations:** 1 Laboratoire Modélisation, Epidémiologie et Surveillance des Risques Sanitaires (MESuRS), Conservatoire National des Arts et Métiers, Paris, France; 2 Unité PACRI, Institut Pasteur, Conservatoire National des Arts et Métiers, Paris, France; 3 Endemic Medicine Department, Faculty of Medicine, Helwan University, Cairo, Egypt; 4 Tropical Medicine Department, Faculty of Medicine, Fayoum University, Fayoum, Egypt; 5 MRC Centre for Global Infectious Disease Analysis, Department of Infectious Disease Epidemiology, Imperial College London, London, UK; 6 Institut Pasteur, Epidemiology and Modelling of Antibiotic Evasion (EMAE), Paris, France; 7 Université Paris-Saclay, UVSQ, Inserm, CESP, Anti-Infective Evasion and Pharmacoepidemiology Team, Montigny-Le-Bretonneux, France; 8 IAME, UMR 1137, Université Paris 13, Sorbonne Paris Cité, Paris, France; 9 Present Address: iEES Paris, Sorbonne Université, Campus Pierre et Marie Curie, 4 Place Jussieu, 75005 Paris, France

**Keywords:** Infectious diseases, Applied mathematics

## Abstract

In response to the COVID-19 epidemic, Egypt established a unique care model based on quarantine hospitals where only externally-referred confirmed COVID-19 patients were admitted, and healthcare workers resided continuously over 1- to 2-week working shifts. Using a mathematical model accounting for the false-negative rates of RT-PCR tests, we computed the incidence rate of SARS-CoV-2 infection among HCWs, while unveiling the proportion of infections remaining undiagnosed despite routine testing. We relied on longitudinal data, including results of routine RT-PCR tests, collected within three Egyptian quarantine hospitals. We estimated an incidence rate (per 100 person-day, PD) of 1.05 (95% CrI 0.58–1.65) at Hospital 1, 1.92 (95% CrI 0.93–3.28) at Hospital 2 and 7.62 (95% CrI 3.47–13.70) at Hospital 3. We found that the risk for an HCW to be infected during a working shift lay within the range of risk levels previously documented in standard healthcare settings for Hospitals 1–2, whereas it was > threefold higher for Hospital 3. This large variation suggests that HCWs from quarantine hospitals may face a high occupational risk of infection, but that, with sufficient infection control measures, this risk can be brought down to levels similar to those observed in standard healthcare settings.

## Introduction

Healthcare settings have faced multiple challenges related to the currently ongoing severe acute respiratory syndrome coronavirus 2 (SARS-CoV-2) pandemic. They have notably had to deal with influxes of infected patients across successive epidemic waves while controlling the risk of nosocomial spread to other patients and staff. As a result, healthcare workers (HCWs) have been a population of interest in terms of risk assessment and control measure implementation since early stages of the pandemic^1,2,3^. Indeed, HCWs have been found to face a higher incident risk of infection than the general community^4^ and other essential occupations^5^. As of the beginning of May 2020, HCWs represented about 4% of all reported patients with coronavirus disease 2019 (COVID-19), worldwide^6^. In addition, the high proportion of asymptomatic SARS-CoV-2 infections^7,8^ and thus, potentially infected yet undetected HCWs represented a particular challenge in the control of nosocomial SARS-CoV-2 transmission.

Egypt was early identified as one of the African countries most vulnerable to SARS-CoV-2 importation^9^. On February 14, 2020, Egypt reported the first confirmed case of COVID-19 in Africa and remained among the five African countries most impacted by the COVID-19 epidemic up to the end of 2020^10^. From February 14 to August 31, 2020 (the period to which we refer to as the ‘first wave*’* of the Egyptian epidemic), the COVID-19 epidemic in Egypt resulted in about 9700 confirmed infections and 5500 deaths reported nationally^11^. These numbers most certainly reflect underreporting of the real number of infections, due to the high proportion of asymptomatic SARS-CoV-2 infection and limited testing.

To mitigate the high risk of SARS-CoV-2 spreading, Egypt established a unique care model under supervision from the World Health Organization, whereby specific hospitals were assigned as quarantine hospitals for patients with COVID-19, and where dedicated medical teams resided in the hospital during working shifts of various durations^12^. The quarantine-hospital protocol required HCWs to be screened for SARS-CoV-2 infection before starting a working shift, and to be systematically tested at the end of a shift, as well as during shifts, in the case of developing symptoms and/or outbreak suspicion.

While the quarantine-hospital strategy has the potential to be highly efficient in terms of patient care, as well as in limiting the potential spread of the virus from hospitals into the community, its impact on infection risk for HCWs remains understudied. One theoretical modelling study assessing healthcare workforce organization found that alternating HCW teams by 1-week periods may reduce the overall number of infected HCWs^13^. However to the best of our knowledge, previous epidemiological studies focusing on the infection risk faced by Egyptian HCWs have been conducted exclusively in non-quarantine settings^14–17^. Yet, the quarantine strategy was adopted again to face the second wave of the COVID-19 epidemic at the national level, and could be adopted for future epidemic waves in Egypt and/or in other countries^18^.

Here, we used mathematical modelling to estimate the risk of SARS-CoV-2 infection among HCWs participating in quarantine-hospital interventions, relying on detailed longitudinal data collected in three Egyptian healthcare facilities (hereafter denoted by Hosp1, Hosp2 and Hosp3) during the first wave of the COVID-19 epidemic.

## Results

The study period of the quarantine-hospital intervention covered ten 2-week shifts in Hosp1, nine ~ 2-week shifts in Hosp2 and five 1-week shifts in Hosp3, during the first COVID-19 wave in Egypt (March–July, 2020). The mean (min–max) number of HCWs per shift was 46 (34–63) in Hosp1, 15 (5–26) in Hosp2 and 19 (16–20) in Hosp3 (Table 1). Over a total follow-up of 8,733 person-days (PD), 54 SARS-CoV-2 infections were observed (i.e., confirmed SARS-CoV-2 infection diagnosis) among 722 HCWs showing no evidence of SARS-CoV-2 antibodies at the beginning of their shifts, across the three hospitals. This represented an overall incidence rate of 0.62 (95% CI 0.45–0.78) diagnosed SARS-Cov-2 infections per 100 PD (Table 2). A significantly higher incidence rate was observed for HCWs working at Hosp3 as compared to the other two hospitals; and HCWs working in intensive care units (ICU) tended to face a lower risk than HCWs working in non-ICU units, though this difference was not statistically significant (Table 2).

**Table 1:** Hospital characteristics.

	Hosp1	Hosp2	Hosp3
Quarantine organization period	March 14–August 1	April 1–July 31	June 6–July 11
Location	Cairo	Fayoum	Cairo
Mean daily number of patients (min–max)	62 (0–108)	37 (0–103)	8 (0–20)
Mean number of HCWs per shift (min–max)	46 (34–63)	15 (5–26)	19 (16–20)
Shift duration	14 Days	7–14 Days	7 Days

Quarantine organization period, location and mean number of HCWs and patients by hospital (Hosp1, Hosp2 and Hosp3).

*HCWs* healthcare workers, *PD* person-days.

**Table 2:** Observed risk of SARS-CoV-2 infection among HCWs, by hospital and by hospital unit.

	Observations		Crude rates		Adjusted Poisson regression		
	Events	PD	Rate per 100 PD	95% CI	IRR	95% CI	${\text{p}}$
**Hospital**							
Hosp1	28	6258	0.45	0.28–0.61	1	Ref	–
Hosp2	11	1808	0.61	0.25–0.97	1.49	0.74–2.87	0.28
Hosp3	15	667	2.25	1.1–3.39	5.59	3.13–10.01	< 0.001
**Hospital unit**							
ICU	15	2628	0.57	0.28–0.86	1	Ref	–
Non-ICU	39	6105	0.64	0.44–0.84	1.49	0.85–2.59	0.16

A total of 54 infections were observed (i.e., diagnosed before or at the end of a shift) over 8733 person-days, in the three hospitals (Hosp1, Hosp1 and Hosp3). Crude rates of SARS-CoV-2 infection among HCWs by hospital and by hospital unit: intensive and non-intensive care units and 95% confidence intervals. A Poisson regression adjusted by hospital and by hospital unit was performed on the observed infections to obtain the incidence rate ratios and 95% confidence intervals.

*CI* confidence interval, *ICU* intensive care unit, *IRR* incidence rate ratio, *PD* person-days.

**Figure 1:**
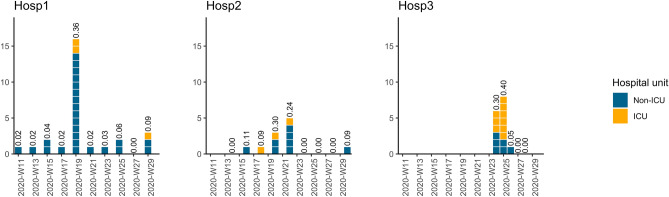
Per-shift number of infections by hospital unit. For a given working shift, healthcare workers were assigned to either the intensive care unit (ICU, yellow) or a non-ICU (blue). Working shifts were 2-week-long in Hosp1 and Hosp2, and 1-week-long in Hosp 3. Dates are given in weeks, denoted by the prefix W followed by the week number of year 2020. Hosp1 was established as a quarantine hospital from March 14th to August 1st (W11 to W31), Hosp2 from April 1st to July 31th (W14 to W31) and Hosp3 from June 6th to July 11th (W23 to W28). In Hosp1 and Hosp2, most infections occurred in non-ICU units. Per-shift attack rates are shown above the bars. Of note, only previously infected HCWs worked during the last two shifts (W27 and W28) in Hosp3.

Healthcare-associated outbreaks among HCWs (observed attack rates of $\ge 20\%$) represented ~ 70% (38/54) of all SARS-CoV-2 infections observed over the study period (Fig. 1). Two of these outbreaks occurred in Hosp2 with 30% (3/10) and 24% (5/21) of HCWs being infected over two different working shifts. An outbreak where 36% of HCWs were infected (16/44) occurred in Hosp1 around the same time period. Two outbreaks took place later on in Hosp3, leading to, respectively, 30% (6/20) and 40% (8/20) of susceptible HCWs being infected over two different shifts. Of note, each outbreak resulted in infections in both ICU and non-ICU. For the last shift in Hosp3, staff recruitment relied exclusively on HCWs who had been infected during the first or the second shifts. This likely prevented HCWs in Hosp3 from becoming infected during this shift (cf. Fig. 1).

The main outcomes estimated by our mathematical model are the mean incidence rates of both observed (i.e., diagnosed) and unobserved (i.e., undiagnosed) SARS-CoV-2 infections in each hospital, over the whole study period. We found significantly different risk levels between the three hospitals (Kolmogorov–Smirnov test), with an incidence rate of 1.05 (95% CrI 0.58–1.65) per 100 PD in Hosp1, 1.92 (95% CrI 0.93–3.28) per 100 PD in Hosp2 and 7.62 (95% CrI 3.47–13.70) per 100 PD in Hosp3.

The probability for a HCW to be infected at the end of a working shift was estimated at 13.7% (95% CrI 7.8–20.8%) and 23.8% (95% CrI 12.2–37.3%) for 2-week shifts at Hosp1 and Hosp2, respectively, whereas a much higher probability of 42.6% (95% CrI 21.9–64.4%) was found for a 7-day shift at Hosp3 (Table 3). These probabilities become 7.1% (95% CrI 4.0–11.0%) and 12.7% (95% CrI 6.3–20.8%), for Hosp1 and Hosp2, respectively, if 7-day shifts are considered as well, for easier comparison (Table 3).

**Table 3:** Model-based estimates of all SARS-CoV-2 infections.

	Median estimated incidence rate per 100 PD (95% CrI)			Probability of infection for a 7-day shift (95% CrI)	Median number of SARS-CoV-2 infections (95% CrI)		
	ICU	Non-ICU	Overall		All	Diagnosed	Proportion of undiagnosed infections
Hosp1	0.59 (0.11–1.61)	1.27 (0.68–2.01)	1.05 (0.58–1.65)	7.1% (4.0–11.0%)	59 (45–74)	31 (15–51)	46.4% (18.8–66.7%)
Hosp2	1.33 (0.33–3.33)	2.75 (1.12–5.02)	1.92 (0.93–3.28)	12.7% (6.3–20.8%)	31 (14–52)	17 (7–31)	45.0% (5.6–70.8%)
Hosp3	9.37 (3.48–19.66)	7.12 (2.06–18.71)	7.62 (3.47–13.70)	42.6% (21.9–64.4%)	41 (19–63)	16 (6–30)	59.2% (34.8–78.8%)

Model-based estimates of the incidence rate, probability of infection for a 7-day working shift and estimated number of SARS-CoV-2 infections in each hospital, over the whole study period. Running our model for each type of care unit (ICU and non-ICU) in each hospital unveils the difference in the risk faced by HCWs between the three hospitals of our study. Indeed, HCWs working in Hosp1 and Hosp2 face a higher risk when assigned to non-ICU, as compared with those working in the ICU; whereas HCWs in Hosp3 face a slightly higher risk when assigned to the ICU. All estimates concern both diagnosed and undiagnosed infections (all), unless otherwise stated (diagnosed).

*CrI* credibility interval, *ICU* intensive care unit, *PD* person-days.

The per-hospital incidence rate estimates yield the following model-based median numbers of total SARS-CoV-2 infections: 59 (95% CrI 45–74) at Hosp1, 31 (95% CrI 14–52) at Hosp2 and 41 (95% CrI 19–63) at Hosp3 (Table 3), which unveils a proportion of undiagnosed infections among HCWs of 46.4% (95% CrI 18.8–66.7%), 45.0% (95% CrI 5.6–70.8%) and 59.2% (95% CrI 34.8–78.8%), respectively.

We further estimated the incident risk by hospital unit. Given the notable differences between hospitals (Fig. 1), we did not assess infection risk by care unit across hospitals, but within each hospital. This confirmed a notable difference between Hosp1–2 and Hosp3: our results suggest that HCWs working in Hosp1 and Hosp2 faced a higher risk when assigned to non-ICU, as compared to those assigned to the ICU; whereas HCWs in the ICU of Hosp3 faced higher risk (see Fig. 1, Table 3).

## Discussion

Here, we studied the risk of incident SARS-CoV-2 infection among HCWs residing in quarantine hospitals, relying on detailed longitudinal data collected during the first wave of COVID-19 in Egypt. We found that most diagnosed infections (70%) occurred during what we defined as healthcare-associated outbreaks (as compared to isolated infections). We observed high variability in nosocomial incidence, ranging from 0.45 to 2.25 infections per 100 PD across the three hospitals. Using a model-based approach, we estimated the risk of both diagnosed and undiagnosed SARS-CoV-2 infections among HCWs, by hospital and by hospital unit (ICU and non-ICU). We further estimated that a substantial proportion of infections may have remained undetected, ranging from 45.0 to 59.2% across the three hospitals.

Our study design has several limitations. First, HCWs were screened using rapid serological tests (RST) before starting their working shifts. This may have allowed recently infected—and probably infectious—HCWs to start a working shift, as antibodies are detected by RST in less than 40% of infected individuals within 7 days since symptoms onset^19^. In addition, a study screening asymptomatic HCWs found a low sensitivity in identifying virus carriers for RST, and thus, they were recommended to be used in association with reverse transcription-polymerase chain reaction (RT-PCR) tests^20^. However, the Egyptian public health authorities’ choice of a protocol relying on rapid serological tests before working shifts was mainly driven by material constraints. Indeed, the use of serological tests as a diagnostic tool was and remains of particular interest in limited-resource contexts due to their lower cost, the minimal equipment required, and faster results, as compared with RT-PCR tests. For our study, this may have led us to slightly underestimate the infection risks, as some HCWs may not actually have been at-risk.

Second, the data collected for this study was scarce. The need to introduce control measures under limited resources forced Egyptian hospitals to implement the quarantine-hospital strategy rapidly and, in some cases, during very short periods of time. Scarce data limits the strength and reach of our methods, and thus, our modeling choices were driven by parsimony. In particular, we assumed a constant overall risk of infection among HCWs over time, in each hospital unit, even though this disregards dynamic fluctuations in risk driven by variation in the number of HCWs and/or patients in the hospitals, as well as the frequency and nature of HCWs’ contacts during their working shifts. Indeed, explicitly modelling different transmission routes (patient-to-HCW vs HCW-to-HCW) would have required additional parameters in the model, leading to identifiability issues due to the scarcity of our data. In addition, this assumption allowed us to compare our results between hospitals and with existing literature on similar contexts or settings (see below). We would like to however stress the importance of developing modelling tools applied to low- and middle- income contexts, despite the difficulties that may come with limited resources. In particular, it is of great interest to evaluate what was the impact of interventions such as the quarantine-hospitals, which was implemented only in a few settings, to accompany future decision-making.

We found a ~ fivefold higher observed risk for Hosp3 as compared with the risk found in Hosp1; whereas the risk of infection at Hosp2 was only slightly higher. The higher risk of SARS-CoV-2 infection we report for Hosp3 and the higher risk faced by HCWs assigned to the ICU of Hosp3 may be partly explained by the short period over which this hospital adopted the quarantine organization, which coincided with the country's highest epidemic activity of the first wave^4^. This may have led to a high proportion of severe and thus highly contagious COVID-19 patients referred to quarantine hospitals together with a higher workload for HCWs. In contrast, Hosp1 adopted the quarantine organization in mid-March 2020, and thus experienced several weeks of lower epidemic intensity that may have improved preparedness for intense COVID-19 activity and implementation of infection control measures for invasive procedures (e.g., intubation in the ICU).

Third, despite the quarantine strategy being adopted nationally, the differences between the organizations of each of the three hospitals (working shifts lengths, quarantine-organization period) should be accounted for when comparing risk estimates across the three hospitals. Data from other hospitals that have adopted a quarantine strategy will be beneficial to better understand occupational risks and eventually study the performance of such a strategy in comparison to standard care settings.

Our model-based approach, accounting for false-negativity rates of testing, as well as the right-truncation of our data, allowed us to estimate the risk of both diagnosed and undiagnosed SARS-CoV-2 infection among HCWs, by hospital and by hospital care unit. We estimated that a rigorous quarantine organization with systematic testing of HCWs at the end of their working shifts captures only about half of all HCW infections. Of note, the higher proportion of infections that remained undetected in Hosp3 (59.2%, vs. 46.4% and 45.0% in Hosp1 and Hosp2, respectively) may be explained by the shorter duration of the working shifts (7 days), which may result in false-negative tests for HCWs infected a short time before the end of their shift. Moreover, these results suggest that in the absence of systematic testing at the end of quarantine working shifts, an even larger proportion of infections among HCWs may remain undetected, thus putting HCWs’ close contacts at risk of infection^21^ and, consequently, putting themselves at risk for adverse psychological symptoms^22^.

The model-based estimates we found for the SARS-CoV-2 infection risk in Hosp1 and Hosp2 are consistent with infection point-prevalence reported in earlier studies performed in non-quarantine Egyptian hospitals (varying from 4.2 to 14.3%) and with incidence estimates reported among front-line HCWs in the UK (13% infection rate after one month)^15,16,17,23^. For comparison, a summary of previous results obtained early in the COVID-19 pandemic and/or specifically in the Egyptian context is presented in Table 4. Moreover, our results suggest that HCWs assigned to non-ICU face a higher risk than those assigned to the ICU, in Hosp1 and Hosp2, which is in line with that observed in previous studies addressing the occupational risk of SARS-CoV-2 infection for HCWs in non-quarantine settings^24^. Overall, our findings on Hosp1 and Hosp2 suggest that, providing sufficient preparedness, HCWs working in quarantine hospitals may not face a higher infection risk, and thus highlight the benefits of implementing a quarantine-hospital strategy.

**Table 4:** Our results in context.

Indicator	Estimate (%)	Context	Study
Per-shift probability of infection (95% CrI)	12.8 (7.6–19.5)	Hosp1 (14-days shift)	Our results
	17.3 (7.5–30.7)	Hosp2 (14-days shift)	
	48.2 (23.8–74.5)	Hosp3 (7-days shift)	
Point-prevalence	4.2	Symptomatic and asymptomatic HCWs in 12 healthcare facilities (N = 4040)	^16,17^
Point-prevalence	14.3	Asymptomatic HCWs in emergency departments of tertiary care facility (N = 203)	^15^
Incidence rate	13.0	Front-line HCWs testing negative at enrollment	^23^

Comparison of the per-shift probability of infection estimated in Hosp1–3 with earlier estimates of the point-prevalence in other Egyptian studies and the observed cumulative incidence rate among HCWs in an English study.

A notable strength of our study lies in the specific nature of the quarantine hospital set-up: because HCWs resided continuously in the hospitals over their entire working shifts, we were able to exclude risk of infection in the community and specifically quantify the nosocomial risk for HCWs. Conversely, results reported from non-quarantine hospitals worldwide are generally unable to distinguish between the nosocomial vs. community risk of infection. However, as most studies conducted in healthcare settings, we were unable to distinguish between patient-to-HCW and HCW-to-HCW routes of transmission. A previous study on several nosocomial COVID-19 healthcare-associated outbreaks in Germany reported that HCW-to-HCW transmission could represent an outsized risk as compared to the one due to infected patients^25^. Still, assessing the relative contribution between patient-to-HCW versus HCW-to-HCW SARS-Cov-2 transmission was left for future work. However, it is worth noting that during the outbreaks observed in our study, infections occurred in different care units, which may be indicative of HCW-to-HCW transmission, rather than simultaneous independent events of patient-to-HCW transmission. In early stages of the pandemic, personal protective equipment (PPE) placed a focus on the risk induced by patients, at times underestimating the risk of infection from—infected and undiagnosed—colleagues. Indeed, modeling studies based on non-quarantine hospitals settings have found that the contribution of HCW-to-HCW transmission may exceed that of patient-to-HCW^26,27^. This may be especially true for quarantine hospitals in which HCWs share resting and conviviality rooms for longer times than in standard care settings. In addition, although HCWs working at quarantine hospitals have been found to develop less adverse mental health symptoms than HCWS working at diagnosis and triage hospitals, still more than 30% of them have reported moderate to severe symptoms of depression and anxiety disorder^28^. Hence, adapting risk-control measures, such as the use of PPE and social distancing between colleagues, while ensuring sufficient social interaction and support to maintain HCWs’ mental health constitutes a specific challenge for quarantine hospitals^22,28^.

In conclusion, the key role played by healthcare settings during pandemic waves since 2020 worldwide^29^ highlights the need for innovative strategies to control the nosocomial risk. Quarantine hospitals, which effectively isolated non-COVID patients from COVID patients and allowed to closely monitor at-risk HCWs, may provide interesting solutions to this challenge. Our results suggest that, with sufficient anticipation and infection control measures, the risk faced by HCWs working in such quarantine hospitals can be brought down to levels similar to those observed in standard COVID-19 care settings. However, a comprehensive assessment of quarantine hospital care models should also include their impact on HCWs’ mental health as well as the potential benefits of earlier infection diagnosis, which is likely to reduce further hospital, household and community transmission^22^.

## Methods

### Study settings

Data was collected within three Egyptian hospitals located in Cairo (Hosp1 and Hosp3), and Fayoum (middle Egypt, Hosp2), that were temporarily transformed into quarantine hospitals during the first COVID-19 wave. During the quarantine-organization period (Hosp1: March 14th to August 1st, 2020; Hosp2: April 1st to July 31th; Hosp3: June 6th to July 11th, 2020, a fourfold shorter period), only externally-referred COVID-19 confirmed patients were admitted to these hospitals for medical care. Multidisciplinary medical teams fully dedicated to COVID-19 patient management worked in total isolation, organized by shifts (Hosp1: 2-week shifts; Hosp2: 1 to 2-week shifts; Hosp3: 1-week shifts). During their shift, HCWs were assigned either to ICU or non-ICU units. HCWs were screened for SARS-CoV-2 infection before starting a working shift using rapid serological IgM/IgG antibody tests (Artron laboratories Burnaby, Canada; sensitivity: 83.3%, specificity: 100%^30^). Only HCWs with no SARS-CoV-2 antibodies were allowed to start working in the hospital; except for the 5th and last shift in Hosp3, where staff recruitment relied exclusively on HCWs who had been previously infected (positive serological tests), and were additionally tested using RT-PCR tests on nasopharyngeal swabs, obtaining negative results. During the working shifts, HCWs were tested for SARS-CoV-2 infection using RT-PCR tests: (1) routinely at the end of the shift, (2) upon symptoms, and (3) in case of outbreak suspicion (> 2 positive tests among HCWs). HCWs testing negative at the end of the shift were then released for self-isolation at home for two weeks. HCWs testing positive before or during their shift self-isolated at home in the case of presenting no or mild symptoms, or were admitted to the same quarantine hospital for medical care in the case of presenting moderate to severe symptoms.

### Observed risk of SARS-CoV-2 infection

Crude incidence rates were obtained from the number of incident SARS-CoV-2 infections observed (i.e., diagnosed during or at the end of a shift) among HCWs screened as seronegative for SARS-CoV-2 before their working shift. Incidence rate ratios were obtained through a Poisson regression adjusted by hospital and by type of care unit (ICU/ non-ICU). In addition, we computed the attack rates over each working shift, based on the observed infections among HCWs having a negative screening test at the beginning of the working shift. Observed per-shift attack rates of ≥ 20% were considered to be healthcare-associated outbreaks among HCWs.

### Model-based estimates of the incidence rate of SARS-CoV-2 infection

An important feature arising from COVID-19 surveillance within quarantine hospitals is potential right-truncation of data: HCWs infected a short time before the end of a shift are likely to remain undiagnosed despite the systematic testing at the end of shifts, especially if the test is performed early in the incubation period. We thus developed the following mathematical model to estimate the incidence rate of both diagnosed and undiagnosed SARS-CoV-2 infections.

We simulated the daily number of incident infections among HCWs, $X_{k,i}^{A}$ based on an unobserved binomial process:

$$X_{k,i}^{A} \sim Bin\left( {n = S_{k,i}^{A} ,p = \lambda } \right)$$

where $k$ denotes the working shift, $i$ denotes the day within the shift, $A$ is the hospital unit of HCWs’ assignments ($A \in \left\{ {{\text{ICU,}}\;{\text{Non - ICU}}} \right\}$), $S_{k,i}^{A}$ denotes the number of susceptible HCWs assigned to A, at the beginning of the $i$th day of shift $k$ and $\lambda$ denotes the daily probability of SARS-CoV-2 infection faced by HCWs, assumed to be constant over the whole study period in each hospital unit.

The number of susceptible HCWs was updated on a daily basis:

$$S_{k,i + 1}^{A} = S_{k,i}^{A} - X_{k,i}^{A}$$

To model right-truncation in the surveillance data, we simulated an observation process representing the systematic RT-PCR testing of HCWs at the end of each shift, accounting for documented variation in test sensitivity as a function of time since infection^31^. The period of time since infection was computed as $j_{k} - i,$ where $j_{k}$ denotes the day at the end of shift ${\text{k}}$ (i.e., the day when testing was performed) and $i$ denotes the day of infection. Then, the daily number of incident infections eventually diagnosed at the end of the $k$th shift was given by

$$I_{k,i}^{A} = X_{k,i}^{A} \theta_{{j_{k} - i}}$$

where $\theta_{{j_{k} - i}}$ denotes the probability of testing positive to RT-PCR, $j_{k} - i$ days after infection.

We used a Bayesian Markov Chain Monte-Carlo (MCMC) approach to estimate the parameter $\lambda$ that best fitted the observed number of infections. We considered a non-informative uniform prior distribution $\sim U\left( {0,1} \right)$ for $\lambda$ and the following binomial likelihood function:

$$L\left( {\lambda {\text{|data}}} \right) = \mathop \prod \limits_{A} \left( {\begin{array}{*{20}c} {\mathop \sum \limits_{k,i} N_{k,i}^{S,A} } \\ {\mathop \sum \limits_{k,i} N_{k,i}^{I,A} } \\ \end{array} } \right)\left( {\frac{{\mathop \sum \nolimits_{k,i} I_{k,i}^{A} }}{{\mathop \sum \nolimits_{k,i} S_{k,i}^{A} }}} \right)^{{\mathop \sum \limits_{k,i} N_{k,i}^{I,A} }} \left( {1 - \frac{{\mathop \sum \nolimits_{k,i} I_{k,i}^{A} }}{{\mathop \sum \nolimits_{k,i} S_{k,i}^{A} }}} \right)^{{\mathop \sum \limits_{k,i} N_{k,i}^{S,A} - \mathop \sum \limits_{k,i} N_{k,i}^{I,A} }} ,$$

where $\mathop \sum \limits_{k,i} N_{k,i}^{S,A}$ and $\mathop \sum \limits_{k,i} N_{k,i}^{I,A}$ denote the total number of susceptible HCWs (denoted by the superscript $S$) and the total number of HCW infected and diagnosed with COVID-19 (denoted by the superscript ${\text{I}}$), observed in hospital unit $A$, during the study period, respectively. The model was thus fitted to the total number of observed infections in each hospital unit.

Medians and 95% credibility intervals (CrI) for $\lambda$ were computed from posterior samples obtained after 10,000 model runs. The chains were visually inspected for convergence (Supplementary Fig. 1). To compare the resulting distributions for the incident risk, $\lambda$, between two hospitals and/or hospital units, we performed two-sample Kolmogorov–Smirnov tests.

For each hospital, we further estimated the probability for a susceptible HCW to be infected at the end of a shift as

$$P\left( {{\text{Infected}}\;{\text{at}}\;{\text{shift}}\;{\text{end}}} \right) = 1 - \left( {1 - \lambda } \right)^{d},$$

where $d$ is the length of the working shift, in days.

Finally, to assess the proportion of undetected infections among HCWs, we ran our model on each hospital, using the posterior distributions for $\lambda$, to obtain an estimate for the real number of SARS-CoV-2 infections in each hospital (both diagnosed and undiagnosed), over the study period.

The numeric implementation of the model was coded in R 4.0.3^32^, using the FME package^33^ for parameter estimation. The code is available at https://github.com/sjijon/SARS-CoV-2_QuarantineHospitals.

### Ethical statements

This study was approved by the Research Ethics Committee (REC) of the Central Directorate of Research and Health Development and Reviews at the Egyptian Ministry of Health and Population (Serial: 25-2020/16), and by REC for human subject research at the Faculty of Medicine, Helwan University (Serial: 50-2020). All methods were performed in accordance with the relevant guidelines and regulations.

In addition, all participant healthcare workers provided informed consent during enrolment.

## Supplementary Information

Supplementary Information.

## Data Availability

Data (in aggregate form) and codes are available at: https://github.com/sjijon/SARS-CoV-2_QuarantineHospitals.
