# Supplementary material for: Estimating the risk of incident SARS-CoV-2 infection among healthcare workers in quarantine hospitals: the Egyptian example
Source: Sci Rep. 2022 Nov 17;12:19773. doi: 10.1038/s41598-022-23428-x (PMC9670048; doi:10.1038/s41598-022-23428-x)
Supplement: Supplementary file 1 — Supplementary Information. [file 41598_2022_23428_MOESM1_ESM.pdf]

Supplementary Material

Estimating the risk of incident risk of SARS-CoV-2 infection among healthcare workers residing in Egyptian quarantine hospitals

Sofía Jijón[1,2†] *(PhD), Ahmad Al Shafie*[3†] *(MSc), Essam Hassan*[4]*,* EMAE-MESuRS working group on nosocomial SARS-CoV-2 modeling*, Laura Temime*[1,2] *(PhD), Kévin Jean*[1,2,4]* *(PhD), Mohamed El-Kassas*[3*]*(MD)*

[1] Laboratoire Modélisation, épidémiologie et surveillance des risques sanitaires (MESuRS), Conservatoire national des Arts et Métiers, Paris, France

[2] Unité PACRI, Institut Pasteur, Conservatoire national des Arts et Métiers, Paris, France

[3] Endemic Medicine Department, Faculty of Medicine, Helwan University, Cairo, Egypt

[4] Tropical Medicine Department, Faculty of Medicine, Fayoum University, Fayoum, Egypt

[5]MRC Centre for Global Infectious Disease Analysis, Department of Infectious Disease Epidemiology, Imperial College London, United Kingdom

Supplementary figures

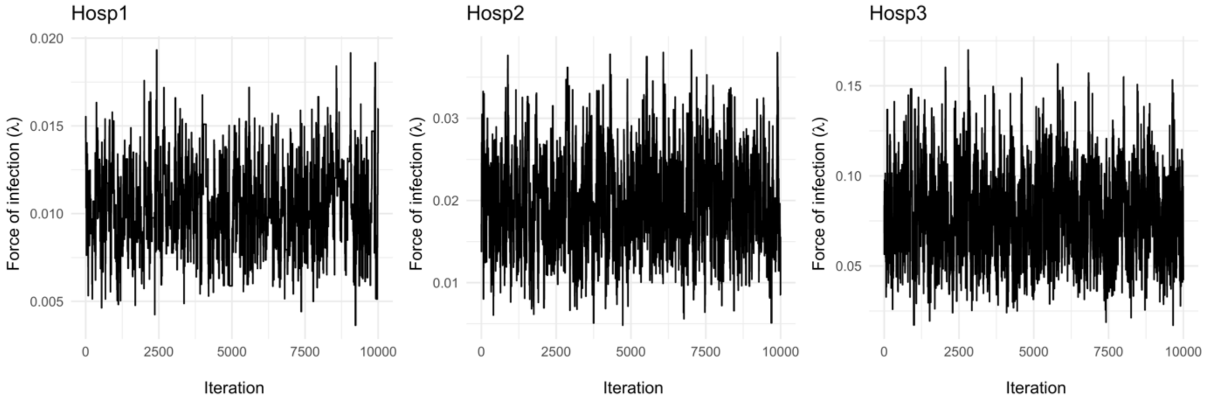

Figure S1 Markov Chain Monte-Carlo diagnostics
Trace plot of the posterior distribution for the force of infection (λ), for Hosp1–3. In 10,000 iterations, the sample space is explored many times and no high serial correlation is apparent in the chain.
